# Noval Dual-Emission Fluorescence Carbon Dots as a Ratiometric Probe for Cu^2+^ and ClO^−^ Detection

**DOI:** 10.3390/nano11051232

**Published:** 2021-05-07

**Authors:** Jiaqing Guo, Aikun Liu, Yutian Zeng, Haojie Cai, Shuai Ye, Hao Li, Wei Yan, Feifan Zhou, Jun Song, Junle Qu

**Affiliations:** 1 Key Laboratory of Optoelectronic Devices and Systems, Center for Biomedical Optics and Photonics (CBOP), College of Physics and Optoelectronic Engineering, Shenzhen University, Shenzhen 518060, China; ling-chen3344@163.com (J.G.); akl0308@163.com (A.L.); cengyutim@163.com (Y.Z.); 2070456098@email.szu.edu.cn (H.C.); yanwei930@163.com (W.Y.); zhoufeifan1982@hotmail.com (F.Z.); songjun@szu.edu.cn (J.S.); jlqu@szu.edu.cn (J.Q.); 2 Moscow Engineering Physics Institute, National Research Nuclear University, MEPhI, 115409 Moscow, Russia

**Keywords:** carbon dots, ratiometric fluorescence, ions detection

## Abstract

The use of carbon dots (CDs) with dual emission based on ratiometric fluorescence has been attracting attention in recent times for more accurate ion detection since they help avoid interference from background noise, probe concentration, and complexity. Herein, novel dual-emission nitrogen-doped CDs (NCDs) were prepared by a simple method for Cu^2+^ and ClO^-^ detection. The NCDs showed excellent anti-interference ability and selectivity for different emissions. In addition, a good linear relationship was observed between the fluorescence intensity (FI) of the NCD solutions in different emissions with Cu^2+^ (0–90 μM) and ClO^−^ (0–75 μM). The limits of both Cu^2+^ detection and ClO^−^ were very low, at 17.7 and 11.6 nM, respectively. The NCDs developed herein also showed a good recovery rate in water for Cu^2+^ and ClO^−^ detection. Hence, they are expected to have a more extensive application prospect in real samples.

## 1. Introduction

Carbon dots (CDs) are widely used for ion detection [1,2,3], biological imaging [4,5], and disease diagnosis and treatment [6,7] because of their excellent optical properties, good biocompatibility, low biotoxicity, and low cost [8,9,10]. Among these, ion detection is the most common application of CDs, and good results have been achieved for actual sample detection with CDs. CDs are particularly useful for detecting ions because of their good selectivity [11,12,13], low detection limit [14,15], and wide detection range [16,17,18], and hence, they are attracting the increasing interest of the research community. However, the vast majority of CDs used for ion detection display only single emission, which makes the detection system susceptible to background noise and probe concentration, as well as the complexity of the real sample. As a result, the accuracy and application value of the experimental results greatly decrease. Therefore, a new ion detection method needs to be developed that can be used more widely in real-life samples and provide more accurate detection results.

Recently, dual-emission CDs based on ratiometric fluorescence have been used to develop novel methods for more accurate ion detection [19,20,21,22,23,24,25,26,27]. The mechanism depends on the fluorescent properties of the dual-emission CDs. The two different emission centers of these CDs show different levels of sensitivity to ions. The exact content of the detected ions can be calculated by establishing a proportional relationship between the fluorescence intensities of different emission centers. Due to the built-in correction of two distinct emission centers, this method can effectively reduce background interference. Some novel ratiometric CDs have been used as probes to detect different ions. Zhu et al. prepared dual-emission CDs centered at 485 and 644 nm [28]. The fluorescence peak of the CDs at 485 nm was gradually quenched by Cu^2+^, while that at 644 nm maintained its original intensity throughout. Thus, these CDs could be used as a ratiometric fluorescent sensor for Cu^2+^ ions. Some ratiometric probes can be used to detect different ions simultaneously [29,30,31,32]. Wang et al. carbonized glutathione in a water/formamide mixture to prepare novel dual-emission CDs [33], which displayed two emissions at 470 and 655 nm. The CDs of the two ions did not interfere with each other; when Fe^3+^ was added to the CD solutions, the emission center of the CDs at 655 nm displayed a clear quenching. Similarly, when Zn^2+^ was added to the CD solutions, the emission center of the CDs at 470 nm displayed an obvious quenching effect. Thus, the CDs can be used as a good dual-ratiometric probe for simultaneous detection of Zn^2+^ and Fe^3+^.

Although dual-ratiometric probes have significant advantages, very few such probes can accurately detect the common ions. Therefore, it is necessary to develop suitable dual-ratiometric probes for the accurate detection of common ions. This work explores novel dual-ratiometric nitrogen-doped carbon dots (NCDs) and uses them to effectively and simultaneously detect Cu^2+^ and ClO^−^.

## 2. Experimental Section

### 2.1. Chemicals

For this study, 2,4-dihydroxybenzoic acid and glycine were supplied by Macklin (Shanghai, China). Metal salts (NaClO, CuSO_4_, MnCl_2_·4H_2_O, NaSO_3_, KH_2_PO_4_, Pb(NO_3_)_2_, (CH_3_COO)_2_Zn, NaF, KCl, NaBr, NaHCO_3_, BaCl_2_·2H_2_O, AgNO_3_, Na_2_H_2_PO_2_, Ni(NO_3_)_2_·6H_2_O, CaCl_2_, MgCl_2_·6H_2_O, CrCl_3_·6H_2_O), and other chemicals such as K_2_CO_3_, NaOH, and HCl were supplied by Macklin (Shanghai, China). All raw chemicals were of analytical grade and were used without further purification. Ultrapure water was prepared using a Milli-Q system (Millipore, Burlington, MA, USA).

### 2.2. Instruments

The microstructure and optical properties of the NCDs were investigated. Transmission electron microscopy (TEM) was performed using FEI Tecnai G2 F20 to observe the morphologies of the NCDs. The chemical composition of the NCDs was analyzed by XPS (Thermo ESCALAB 250Xi). Fourier transform infrared spectroscopy (FT–IR, Nicolet 5700 spectrometer, Thermo Electron Corp, Waltham, MA, USA) was used to identify the chemical structures of the NCDs. Ultraviolet–visible (UV–vis) spectroscopy (UV-2550 Shimadzu, Kyoto, Japan) and fluorescence spectroscopy (Varian Cary Eclipse Agilent, USA) were used to determine the optical properties of the NCDs.

### 2.3. Synthesis of NCDs

First, 2,4-dihydroxybenzoic acid (2 g), glycine (1 g), and ultrapure water (40 mL) were weighed and placed in a Teflon-lined autoclave. Next, the mixture was heated to 200 °C for 24 h, and the primary product was obtained after allowing the mixture to cool naturally to room temperature. Finally, the NCD solutions were obtained by performing dialysis for 3 days and lyophilized to obtain the final NCD powders.

### 2.4. Specificity of Cu^2+^ and ClO^−^ Detection

To elucidate the specificity of the NCDs as probes for ion detection, different ion solutions (2 mM) and NCD solutions (1.0 mg/mL) were added to a 5 mL colorimetric tube. The following ions were used to make the ion solutions: ClO^−^, Cu^2+^, Mn^2+^, SO_3_^2-^, K^+^, H_2_PO_4_^−^, NO_3_^−^, Zn^2+^, F^−^, Cl^−^, Br^−^, HCO_3_^−^, Ba^2+^, Pb^2+^, Ag^+^, H_2_PO_2_^−^, Ni^2+^, Ca^2+^, K^+^, Mg^2+^, SO_4_^2−^, AC^−^, Cr^3+^, and CO_3_^2−^. Their effect on the FI of NCDs was observed at 311 and 497 nm under 458 nm and 515 nm excitation sources, respectively. All experiments were performed more than thrice.

### 2.5. Determination of the Standard Curve for ClO^−^ and Cu^2+^ Detection

ClO^-^ and NCD solutions were added in different concentrations (0, 5.0, 10.0, 15.0, 25.0, 35.0, 45.0, 55.0, 65.0, and 75.0 μM) to a 5 mL colorimetric tube, and their FI spectra were recorded under 311 and 497 nm excitations. All experiments were performed more than thrice.

Similarly, Cu^2+^ and NCD solutions were added in different concentrations (0, 10.0, 20.0, 30.0, 40.0, 50.0, 60.0, 70.0, 80.0, and 90.0 μM) to a 5 mL colorimetric tube, and their FI spectra were also recorded under 311 and 497 nm excitations. All experiments were performed more than thrice.

### 2.6. Analysis of Real Samples

Real samples (tap water) were first filtered through the 0.22 μm PES water phase membrane. The filtered water samples were then spiked with ClO^−^ and Cu^2+^ to obtain different concentrations of ClO^−^ and Cu^2+^ solution (1.0 μM, 2.0 μM, and 3.0 μM). Then, NCDs were added into each of these solutions, and the content of ClO^−^ and Cu^2+^ in the solutions was quantified by the FI, respectively. All experiments were performed in triplicate.

## 3. Results and Discussion

**Figure 1:**
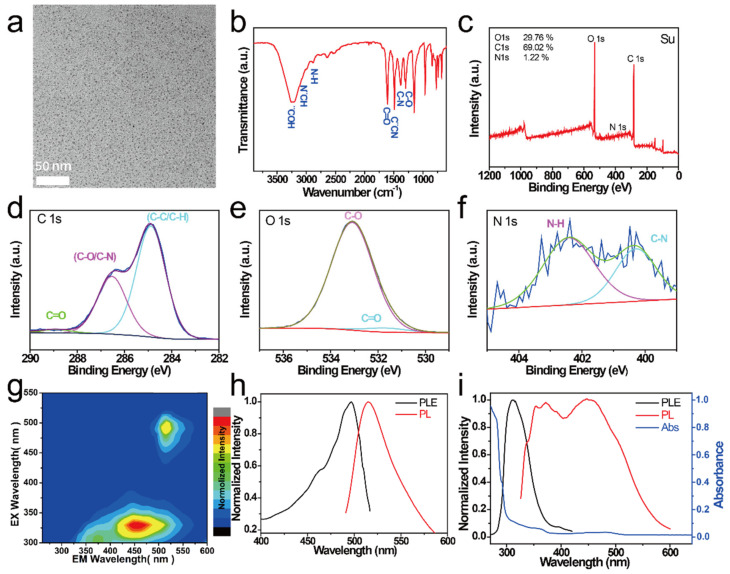
(**a**) TEM image, (**b**) FT–IR spectrum, and (**c**) XPS spectrum of NCDs. (**d**) Corresponding high-resolution C_1s_ spectra, (**e**) O_1s_ spectra, and (**f**) N_1s_ spectra of NCDs. (**g**) Excitation–emission color 3D map of NCDs. (**h**) Excitation (black curve) and fluorescence (FL) spectra (red curve) of NCDs (311 nm excitation). (**i**) Absorption (blue curve), excitation (black curve), and fluorescence (FL) spectra (red curve) of NCDs (497 nm excitation).

The morphology of the as-prepared NCDs was observed using TEM. As shown in Figure 1a, the NCDs possessed a monodisperse spherical structure with uniform sizes. FTIR and XPS were used to determine the chemical composition and surface groups of the NCDs. As shown in Figure 1b, the peaks at 3240 and 3076 cm^−^^1^ in the FT–IR spectrum of the CDs correspond to –OH stretching vibrations and –NH_2_ stretching vibrations, respectively [34,35]. The peaks corresponding to C=O stretching, C=N stretching vibrations, C–N bending modes, C–N bending modes, and C–O stretching vibrations were located at 1606, 1489, 1384, and 1295 cm^−^^1^, respectively. This observation indicated the presence of hydrophilic groups (–OH, –COOH, and –NH_2_) on the surface of the NCDs, which was further confirmed by the XPS spectrum [36,37,38,39,40], as shown in Figure 1c. The XPS spectra of the NCDs showed that they contained C (29.8%), O (69.0%), and N (1.2%) elements. The high-resolution C 1s spectrum of NCDs exhibits four peaks at 284.88, 286.58, and 288.98 eV, corresponding to C-C, C-O/C-N, and C=O, respectively (Figure 1d). The two peaks at 531.08 and 533.48 eV in the high-resolution O 1s spectrum can be assigned to C-O and C=O, respectively (Figure 1e). The high-resolution N 1s spectrum has two peaks at 400.28 eV (C-N) and 402.38 eV (N-H) (Figure 1f).

The optical properties of the NCDs were investigated using fluorescence spectra and UV–visible absorption spectra. The excitation–emission 3D color map of the NCDs is shown in Figure 1g; three luminescence centers can be observed, one each at ~372, ~454, and ~515 nm (Figure 1h,i). The centers at 372 and 454 nm were excited by the 311 nm excitation, while the one at 515 nm was excited by the 497 nm excitation. Figure 1i shows the UV–vis absorption spectra of NCDs, where three peaks at 292 nm (π−π*), 360 nm (*n*−π*), and 478 nm (excitonic absorption band) were clearly observed.

**Figure 2:**
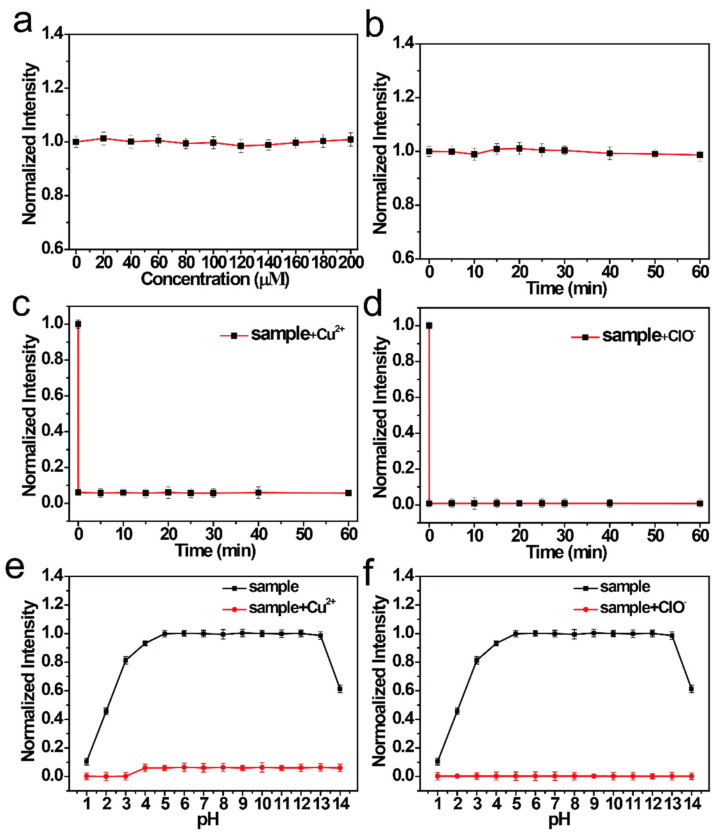
(**a**) Normalized FI of NCDs with different concentrations of NaCl. (**b**) Normalized FI of NCDs at different durations under UV light irradiation. Normalized FI of NCDs (**c**) with Cu^2+^ and (**d**) with ClO^−^ at different response times. Normalized FI of NCDs under different pH values (**e**) with or without Cu^2+^ and (**f**) with or without ClO^−^.

Next, the chemical and optical stabilities of the NCDs were studied. As shown in Figure 2a, no clear change was observed in the FI, which shows that an increase in the concentration of NaCl increases the salt stability of the NCDs. The FI of the NCDs was also detected under UV light (365 nm) exposure (Figure 2b). It was observed that the FI of NCDs changed little with time, implying that NCDs have good stability. When Cu^2+^ was added to the NCDs, the FI rapidly quenched (<1 min) (Figure 2c). The FI similarly quenched quickly when ClO^−^ was added to the NCDs (Figure 2d). These results show that the NCDs are sensitive to both Cu^2+^ and ClO^−^. When Cu^2+^ and ClO^−^ solutions of different pH were added to the NCDs, the FI of the NCDs did not show a significant change in the pH range of 4–14 (Figure 2e). Hence, it can be inferred that NCDs are not affected by the change in pH. After quenching by Cu^2+^ and ClO^−^, the NCD solution shows a good signal response to them between pH of 1 and 14 (Figure 2f).

**Figure 3:**
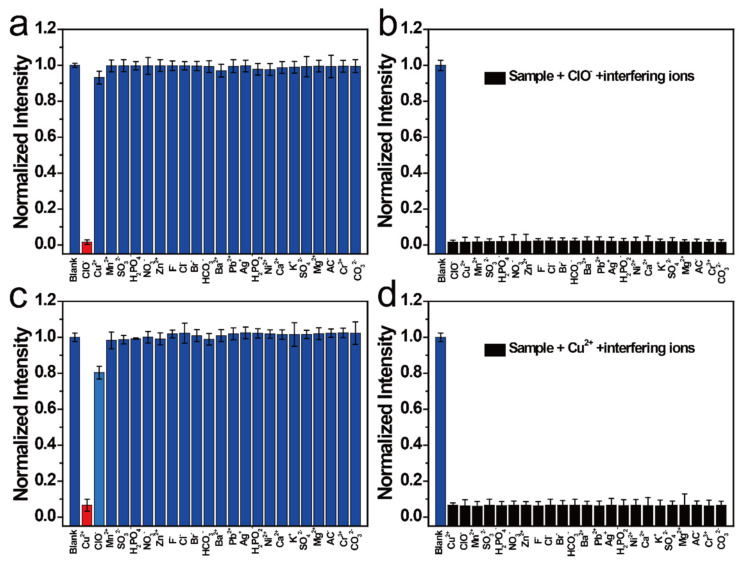
(**a**) Normalized FI of NCDs after the addition of various ions. (**b**) Normalized FI of ClO^−^-NCDs after the addition of various ions. (**c**) Normalized FI of NCDs after the addition of various ions. (**d**) Normalized FI of Cu^2+^-NCDs after the addition of various ions.

Different interfering ions were added to the NCD solution to determine the selectivity and sensitivity of the NCDs. Interestingly, interfering ions barely respond to the NCDs at the 454 nm emission center, except for ClO^-^ (Figure 3a). Similarly, they barely respond to the NCDs at the 515 nm emission center, except for Cu^2+^ (Figure 3c). Hence, it can be suggested that the NCDs can detect Cu^2+^ and ClO^−^ under different excitations. The anti-interference ability of NCDs was also studied at different emission centers; different interfering ions were added to the NCD solution quenched by Cu^2+^ and ClO^−^ (Figure 3b,d, respectively). These data show that NCDs have a good anti-interference ability to detect Cu^2+^ and ClO^−^ in different emission centers.

**Figure 4:**
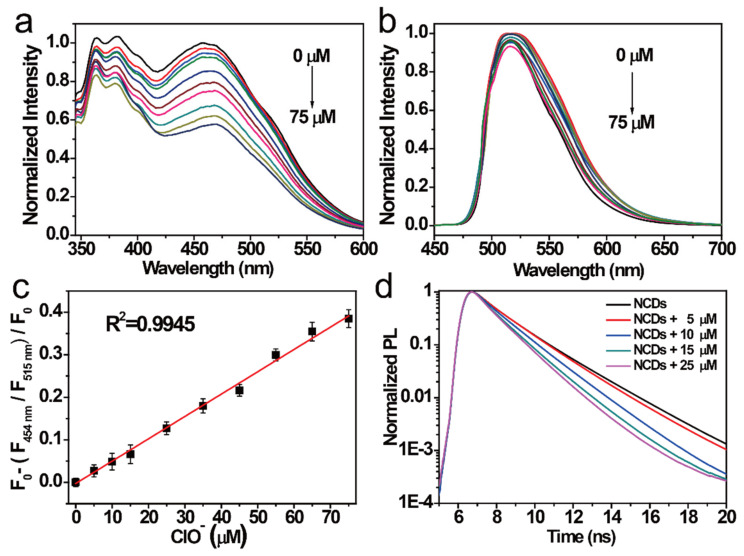
FI of the NCDs excited by (**a**) 311 nm radiation and (**b**) 497 nm radiation after adding different ClO^−^ concentrations (from top to bottom: 0–75 μM). (**c**) Relationship between the F_454 nm/_F_515 nm_ ratio versus the ClO^-^ concentrations (0–75 μM). (**d**) Decay PL spectra of the NCDs quenched by ClO^−^ at different concentrations.

NCDs have good selectivity and sensitivity for the detection of Cu^2+^ and ClO^−^ in different emission centers. Therefore, the effect of ions on the concentration of the NCDs was studied. The FI of NCDs in the 454 nm emission center gradually weakened with increasing concentration of ClO^−^ (Figure 4a). In contrast, the FI of the NCDs in the 515 nm emission center changed a little with the increasing concentration of ClO^−^. Hence, NCDs can be used as a novel ratiometric probe for detecting ClO^−^. Therefore, the relationship between the FI of NCDs in the 454 nm emission center and that of the NCDs in the 515 nm emission center with the same ClO^−^ concentration was further studied. A good linear relationship was observed (Figure 4d). The coefficients (*R*^2^) were estimated to be 0.9945 within 0–75 μM by linear fitting. Based on the “three times the standard deviation” rule [41], the limit of detection (LOD) of this probe is estimated to be 11.6 nM. In Figure 4d, the lifetimes of the NCDs decreased with an increase in the quenching degree. The quenching mechanism of the NCDs by ClO^−^ was studied. When ClO^−^ was added to the NCD solution, the strong oxidation destroyed the two emission centers (372 and 454 nm) of the NCDs and enhanced the nonradiative carrier depopulation, leading to a further weakening of the fluorescence intensity. Compared with other probes reported previously, the NCDs probe demonstrated lower LOD and wider range for ClO^−^ detection [12,14,42].

**Figure 5:**
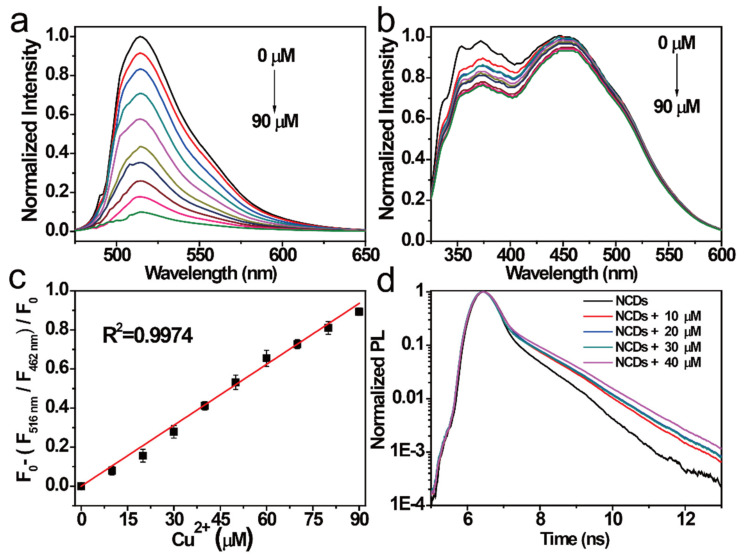
FI of the NCDs excited by (**a**) 497 nm radiation and (**b**) 311 nm radiation after the addition of different Cu^2+^ concentrations (from top to bottom: 0–90 μM). (**c**) Relationship between the F_515nm_/F_454 nm_ ratio versus the Cu^2+^ concentrations (0–90 μM). (**d**) Decay PL spectra of the NCDs quenched by Cu^2+^ at different concentrations.

In addition, the relationships between the FI of NCDs at the 454 nm emission center and that at the 515 nm emission center with the same Cu^2+^ concentrations were studied. The FI of NCDs in the 515 nm emission center gradually weakened with increasing Cu^2+^, whereas that of NCDs in the 454 nm emission center showed a little change with the same increase in Cu^2+^ (Figure 5a,b), which is the opposite of the result obtained with ClO^−^. Thus, NCDs can also be used as a novel ratiometric probe for the simultaneous detection of Cu^2+^. The linear relationship was further established, and the coefficients (*R*^2^) were estimated to be 0.9974 by linear fitting within 0–90 μM. The LOD of this probe was calculated to be 17.7 nM (Figure 5c). The quenching mechanism of the NCDs by Cu^2+^ was investigated by studying the change in the NCD lifetime (Figure 5d). When Cu^2+^ was added to the NCD solution, Cu^2+^ bounded to the surface group of the NCDs by coordination interaction and inhibited the *n*–π* transition of NCDs in the 515 nm emission center. Similar, compared with other probes reported previously, the NCDs probe demonstrated lower LOD and wider range for Cu^2+^ detection [43,44,45].

NCDs have high selectivity, sensitivity, wide detection range, and low detection limit for Cu^2+^ and ClO^−^ detection. Therefore, it can be used as a probe for Cu^2+^ and ClO^−^ detection in water samples. Herein, water samples from a lab (tap water 1) and home (tap water 2) were used. Cu^2+^ and ClO^−^ in different concentrations were added to the NCD solutions in tap water. The concentrations of Cu^2+^ and ClO^−^ in different tap water samples were calculated using the additive recovery method. The above results exhibited a good recovery rate and good accuracy for both Cu^2+^ and ClO^−^ detection (Table 1 and Table 2). Thus, NCDs can be used for ion detection with potential applications in real water samples.

## 4. Conclusions

NCDs were synthesized from 2,4-dihydroxybenzoic acid and glycine via a simple hydrothermal method. The NCDs were found to have high selectivity and sensitivity for Cu^2+^ and ClO^−^ detection in different emissions. In addition, NCDs have a considerably wide detection range and low detection limit for Cu^2+^ and ClO^−^ detection based on ratiometric fluorescence, respectively. These results imply that NCDs can be used for the detection of Cu^2+^ and ClO^−^, with potential applications in real-life water samples.

## Figures and Tables

**Table 1:** Determination of ClO^−^ in tap water samples (*n* = 3).

Sample	Found (μM)	Added (μM)	Total Found (μM)	Recovery (%)	RSD (*n* = 3) (%)
Tap water 1	0.58	1.00	1.56	96.5	2.48
		2.00	2.59	101.7	2.21
		3.00	3.62	106.9	3.63
Tap water 2	0.88	1.00	1.89	101.1	2.89
		2.00	2.88	100.0	1.95
		3.00	3.93	105.7	3.42

**Table 2:** Determination of Cu^2+^ in tap water samples (*n* = 3).

Sample	Found (μM)	Added (μM)	Total Found (μM)	Recovery (%)	RSD (*n* = 3) (%)
Tap water 1	No Found	1.00	0.99	99.0	2.23
		2.00	2.01	100.5	1.65
		3.00	2.95	98.3	1.99
Tap water 2	No Found	1.00	1.04	104.0	2.96
		2.00	1.99	99.5	2.37
		3.00	2.98	99.3	2.18

## Data Availability

The data presented in this study are available on request from the corresponding author. The data are not publicly available due to the author’s readiness to provide it on request.
